# Surface-Associated Plasminogen Binding of *Cryptococcus neoformans* Promotes Extracellular Matrix Invasion

**DOI:** 10.1371/journal.pone.0005780

**Published:** 2009-06-03

**Authors:** Jamal Stie, Gillian Bruni, Deborah Fox

**Affiliations:** 1 Research Institute for Children, Louisiana State University Health Science Center, Children's Hospital, New Orleans, Louisiana, United States of America; 2 Department of Pediatrics, Louisiana State University Health Science Center, Children's Hospital, New Orleans, Louisiana, United States of America; 3 Institute for Microbiology, ETH Zurich, Zurich, Switzerland; Texas A & M University, United States of America

## Abstract

**Background:**

The fungal pathogen *Cryptococcus neoformans* is a leading cause of illness and death in persons with predisposing factors, including: malignancies, solid organ transplants, and corticosteroid use. *C. neoformans* is ubiquitous in the environment and enters into the lungs via inhalation, where it can disseminate through the bloodstream and penetrate the central nervous system (CNS), resulting in a difficult to treat and often-fatal infection of the brain, called meningoencephalitis. Plasminogen is a highly abundant protein found in the plasma component of blood and is necessary for the degradation of fibrin, collagen, and other structural components of tissues. This fibrinolytic system is utilized by cancer cells during metastasis and several pathogenic species of bacteria have been found to manipulate the host plasminogen system to facilitate invasion of tissues during infection by modifying the activation of this process through the binding of plasminogen at their surface.

**Methodology:**

The invasion of the brain and the central nervous system by penetration of the protective blood-brain barrier is a prerequisite to the establishment of meningoencephalitis by the opportunistic fungal pathogen *C. neoformans*. In this study, we examined the ability of *C. neoformans* to subvert the host plasminogen system to facilitate tissue barrier invasion. Through a combination of biochemical, cell biology, and proteomic approaches, we have shown that *C. neoformans* utilizes the host plasminogen system to cross tissue barriers, providing support for the hypothesis that plasminogen-binding may contribute to the invasion of the blood-brain barrier by penetration of the brain endothelial cells and underlying matrix. In addition, we have identified the cell wall-associated proteins that serve as plasminogen receptors and characterized both the plasminogen-binding and plasmin-activation potential for this significant human pathogen.

**Conclusions:**

The results of this study provide evidence for the cooperative role of multiple virulence determinants in *C. neoformans* pathogenesis and suggest new avenues for the development of anti-infective agents in the prevention of fungal tissue invasion.

## Introduction

Invasion of the central nervous system (CNS) by penetration of the blood-brain barrier (BBB) is essential for the establishment of meningoencephalitis by the opportunistic fungal pathogen *Cryptococcus neoformans*, though the mechanism by which this organism invades the BBB has not been definitively established [1]–[4]. Blood-born pathogens may enter the peripheral or CNS compartments through multiple mechanisms of transmigration across the vasculature that include intercellular (paracellular) and transcellular passage, as well as a “Trojan horse” route in which intracellular pathogen is transported across endothelial barriers within host cells [5]. C. *neoformans* is a leading cause of illness and death in persons with predisposing factors including: malignancies, solid organ transplants, AIDS, autoimmune disease, diabetes, corticosteroid use, and pregnancy [6]–[12]. *C. neoformans* is ubiquitous in the environment and enters the lungs by inhalation, with dissemination into the CNS, resulting in a difficult to treat and often-fatal meningoencephalitis. Current drugs used to treat this fungal infection have limited efficacy due to resistance, toxicity, and reduced CNS bioavailability [13]–[16]. Although the immunosuppressed are at greatest risk of infection and death, healthy individuals can also contract the disease due to the widespread presence of the fungus in the environment [17].

The cell wall of *C. neoformans* is critically important for its pathogenesis, as the polysaccharide capsule and melanin are cell wall-associated virulence factors [18]–[22]. The fungal cell wall is unique among cellular organelles in that it provides mechanical strength and acts as a physical barrier to protect the cell from damage. In addition to its structural role, the cell wall is also an important mediator of events necessary for cell-to-cell recognition, growth and morphogenesis, and additionally plays an important role in host immune responses during fungal pathogenesis (reviewed in [23]–[26]). Fungal cell wall proteins also contribute to cell wall organization and stress-induced survival responses by facilitating wall remodeling and signal transduction events within this organelle. Results from recent studies have shown that the cell wall protein repertoire of some fungal pathogens, including *C. albicans*, specifically and directly modulate their capacity to disseminate, in vivo, by subverting the function of soluble host proteins, like plasminogen, and their associated enzyme systems [27]. Compared to other well-characterized fungi, however, the cell wall protein composition of *C. neoformans* is relatively understudied, although several extracellular proteases and immunomodulatory mannoproteins have been identified [28]–[36].

Plasminogen is an abundant protein found in the plasma component of blood and is a central component of the fibrinolytic system that counteracts procoagulative activities to maintain proper flow and multi-system homeostasis. The soluble form of plasminogen within blood plasma exists as an inactive proenzyme that is subject to proteolytic cleavage and activation when cell surface-bound. At its amino terminus, plasminogen consists of five disulfide-linked, homologous repeats that form looped “kringle” structures, and a serine protease catalytic domain at its carboxy terminal end [37]–[40]. The kringle domains of plasminogen structure are ∼80 amino acids in length and mediate the attachment of plasminogen to cell surfaces by binding proteins with accessible carboxyl-terminal or internal lysine residues.

Conversion of plasminogen to the protease, plasmin, is mediated by host-expressed tissue-derived plasminogen activator (tPA) and urokinase (uPA). Plasmin is a broad-specificity serine protease that degrades fibrin and collagen, in addition to other structural proteins. Plasmin also activates other proteolytic enzymes, such as matrix metalloproteinases (MMPs), that degrade the tight junction components of microvascular endothelial cells [41]. This latter function is critical for plasmin-mediated mechanisms of intercellular migration that allow passage of cells across the vasculature into either peripheral tissues or otherwise privileged compartments such as the central nervous system.

The minimal biochemical and structural requirements for plasminogen recruitment predisposes the plasminogen system to pathogen-manipulation for use during invasion of tissue barriers [42]. The relative importance of plasminogen in infectious disease is indicated by the surface-associated plasminogen-binding properties manifested by diverse species of human pathogens, including *Yersina pestis*, *Listeria monocytogenes*, several species of *Streptococcus*, and four pathogenic fungal species, *C. albicans*, *Aspergillus fumigatus*, *Paracoccidioides brasiliensis*, and *Pneumocystis jiroveci (carinii)* [27], [41], [43]–[48]. Several proteins have been found to play a major role in microbial recruitment of plasminogen, including enolase, glyceraldehyde-3-phosphate dehydrogenase (GAPDH), and phosphoglycerate kinase (PGK). These are carbohydrate-active molecules that, though predominantly found in soluble form within the cytosol, are capable of localizing to bacterial and fungal cell walls where they exhibit accessible carboxyl-terminal or internal lysine residues for plasminogen binding [49]. By serving as key surface receptors for plasminogen recruitment, these and other proteins have been shown to function as central mediators of CNS invasion and microbial virulence [44], [46], [49]–[55]. In this report we demonstrate that *C. neoformans* is likewise capable of recruiting plasminogen and activating the plasminogen-fibrinolytic system. Moreover, we find this recruitment process occurs at physiologically relevant plasminogen concentrations and is mediated by similar receptors that include cell wall-localized carbohydrate-active proteins of cytosolic origin.

## Materials and Methods

### Strains and media

The strains used in this study were the following serotype D strains of *C. neoformans*: JEC21 (*MAT*α), JEC20 (*MAT* **a**), B3501A (*MAT*α), FCH78 (*MAT*α *cap59::nat*) and FCH79 (*MAT*α *CAP59 CA59::nat*) and the serotype A (genotype A1/M1) strains C23 and A1 38-2 [56]. All strains were grown in yeast extract-peptone-dextrose (YPD) medium, unless otherwise noted. Sabouraud broth diluted 1∶10 in 1× phosphate-buffered saline (PBS) was used for capsule induction, unless otherwise indicated.

### Disruption of the *C. neoformans CAP59* gene

The *CAP59* gene was disrupted by homologous recombination with a cassette containing the nourseothricin (NAT) resistance marker [57], [58]. The *CAP59* gene (Accession L26508) was amplified from JEC21 genomic DNA with primers FOX124 (5′-CTACGTCGAGCAAGTCAAGG) and FOX125 (5′-ACCTAGGTTGCATGTGTTCC) to generate a 1.5 kbp product (positions 830 to 2298) that was TOPO TA cloned into pYES2.1 (Invitrogen). The NAT cassette was amplified from the pDSF6 plasmid (NAT cassette in pCR2.1) with primers for use in yeast gap repair to introduce 30 base regions of overlap from the *CAP59* gene on either side of a unique Kpn1 site at position 1573 [FOX122 (5′-TCGTCTTCATGAACGATATCTTGCCGCTGCGAGGATGTGAGCTGG) and FOX123 (5′- ATTCAGTGTGGTGGAAGATTTGCGAAGAGAATGTAGAAACTA)]. The Kpn1-linearized CAP59-pYES2.1 plasmid and the amplified NAT cassette were co-transformed into the *S. cerevisiae* strain YPH499 to allow homologous recombination by gap repair and Ura+ transformants were selected for analysis by colony PCR with the flanking FOX 124/125 primer set. Recombinant plasmids were isolated, and the *cap59::nat* disruption cassette was amplified with the flanking primer set and introduced into the serotype D strain JEC21 by biolistic transformation. Disruption of the *CAP59* gene was confirmed by PCR analysis of genomic DNA isolates from comparisons of wild-type (*CAP59*) and nourseothricin-resistant transformants (*cap59::nat*) with the flanking primer set (FOX124/125).

### Plasminogen labeling

Strains were grown at 25°C in YPD to early log (24 hr), log (48 hr) or stationary (72 hr) phases and washed in PBS. Unless otherwise stated, 1×10^8^ cells were labeled with 100 µg purified human plasminogen (Glu-plasminogen, Fitzgerald Industries) in 500 µl PBS with 1.5% BSA at 37°C for 0.5–2 hrs, washed twice in cold PBS/BSA, then either used in additional experiments or analyzed for plasminogen-binding by flow cytometry or Western blot analysis. In some experiments, cells were treated for 30 min at 37°C with carboxypeptidase B (Sigma) or εACA (Sigma) before labeling with plasminogen, after which reactions were further incubated 1 hr (37°C) before washing. For sulfo-NHS-biotin labeling, washed cells were incubated with 1 mg/ml sulfo-NHS-biotin (Pierce) in PBS supplemented with 1 mM MgCl_2_ and 0.01 mM CaCl_2_ for 30 minutes on ice as previously described [59] before or after plasminogen labeling.

### Surface plasminogen activation

Approximately 1×10^8^ log phase cells were washed in PBS and incubated with 100 µg plasminogen for 2–4 hrs at 37°C in PBS with 1.5% BSA, in the presence or absence of 100 ng tissue plasminogen activator (tPA) (Calbiochem) or aprotinin (Roche). After washing four times in PBS to remove unbound plasminogen and tPA, the cells were resuspended in SDS extraction buffer (50 mM Tris-HCl, pH 8.0, 0.1 M EDTA, 2% SDS, 10 mM DTT) and boiled to release surface-associated proteins, which were then fractionated by SDS-PAGE, transferred to PVDF, and analyzed for plasminogen conversion to plasmin by Western blot with anti-plasminogen.

### Flow cytometry

Cells were cultured for 48 hr at 25°C in 50 ml YPD, washed twice in PBS, and suspended at 10^6^ in 500 µl PBS with 0.5% BSA and plasminogen labeled as described above. Plasminogen-specific rabbit antisera (Fitzgerald Industries), was diluted 1∶1000 in a final volume of 100 µl PBS and used to resuspend washed cell pellets. After 1 hr incubation at 25°C, cells were washed twice in ice-cold PBS and labeled with FITC-conjugated goat anti-rabbit IgG secondary antibody (Antibodies Inc) for 30 min on ice then washed as above. Cells were finally resuspended in 0.5 ml PBS before analysis with a FACSVantage SE flow cytometer (BD Biosciences). Data were analyzed with Cell Quest Pro software (BD Biosciences). Because the vast majority of the log phase populations used in this study demonstrated plasminogen-binding activity (≥95%), the data were not gated or otherwise manipulated.

### Invasion assays

Approximately 1×10^8^ log phase cells of strain JEC21 were washed in PBS and incubated with 100 µg plasminogen for 2–4 hrs at 37°C in PBS, in the presence or absence of 100 ng tissue plasminogen activator (tPA) (Calbiochem). After washing four times in PBS to remove unbound plasminogen and tPA, the cells were resuspended in RPMI and added to the upper chamber of BioCoat Matrigel invasion chambers, an in vitro system for the study of cell invasion through basement membrane consisting of BD Falcon™ cell culture inserts containing an 8 µm pore-size PET membrane coated with a uniform layer of BD Matrigel™ Basement Membrane Matrix (BD Biosciences. Organisms were incubated in the upper well of the transwell chamber for 24 hours at 37°C in a hydration chamber prior to analysis of JEC21 penetration through the matrigel. Colony forming units (CFU) per ml were determined by plating dilutions of culture media from the lower well onto YPD, 37°C for 72 hours. At least 4 replicates were used per condition tested.

### Cell wall protein isolation

Cells (serotype D strains) were grown in 50 ml YPD at 25°C for ∼48 hr to log phase. Cell pellets were washed once in PBS and once in sterile ultrapure water. Contaminating capsular polysaccharide was removed by washing cell pellets in dimethyl sulfoxide (DMSO) and incubating at room temperature for thirty minutes, two times. Cell pellets were next washed twice in sterile ultrapure water to remove residual DMSO. Cells were then frozen at −80°C for at least 24 hr and suspended in ice-cold lysis buffer (10 mM Tris-HCl, pH 7.4 with complete protease inhibitor (Roche). Cells were mechanically lysed with 0.5 mm glass beads equal in volume to frozen pellet and agitated on a Mini-beadbeater (Biospec Products) for 12 cycles of 2 min treatment/1 min ice. To insure cell-breakage, the supernatants were transferred to microtubes, placed in an ice/salt/alcohol slurry and probe sonicated using a GE 130 Ultrasonic Processor (Sonics and Materials, Inc) for 10 cycles of 15 sec cavitation at a power setting of 30 W with 2 min off. Cell wall and cytosol fractions were separated from the resulting homogenates by centrifugation at 3000 g (13,000 rpm) for 10 min at 4°C using an AccuSpin Micro R-minifuge (Fisher Scientific), and cell wall fractions were incubated with progressively decreasing concentrations of NaCl solution as described previously [60], [61]. Cell wall proteins were then extracted from cell wall fractions in SDS extraction buffer by boiling at 100°C for 10 minutes. The resulting extracts were clarified at 3000 g (13,000 rpm) and frozen at −80°C prior to lyophilization (Dura-Dry MP Lyophilizer (FTS Systems)). The lyophilized material was quantified by BCA protein assay (Pierce) and stored at −80°C until use. Cell wall proteins obtained by this method were used for identifying plasminogen receptors by 2D PAGE/plasminogen ligand blotting and 1D PAGE after immunoprecipitation with plasminogen conjugated to CNBr-activated Sepharose 4B (Amersham) beads, as described below.

### One- and two-dimensional SDS-PAGE

1D SDS-PAGE was performed using the Novex system (Invitrogen) with NuPAGE 10% bis-tris precast gels. Protein samples were solubilized in NuPAGE LDS sample buffer in 0.005% beta-mercaptoethanol for 5 min at 100°C prior to SDS-PAGE electrophoresis, followed by either protein transfer to PVDF for Western blot analysis or visualization by silver stain [62]. For 2D SDS-PAGE analysis, approximately 150 µg protein sample was precipitated in 10% trichloroacetic acid in acetone at −20°C then washed in acetone and incubated again at −20°C. Samples were dried in a centrivap concentrator (Labconco Corp) and suspended in rehydration sample buffer (8 M urea, 2% CHAPS, 50 mM DTT, 0.2% Bio-Lyte 3/10 ampholyte, 0.001% bromphenol blue (Bio-Rad)) prior to 2D PAGE, as previously described [60]. Duplicate gels were silver stained [62] or transferred to PVDF for plasminogen ligand blotting.

### Plasminogen ligand blotting and Western blot analysis

Plasminogen overlay: 2-D gels were transferred to polyvinylidene difluoride (PVDF) membrane and blocked in PBS, 5% blotto. Blots were incubated with human plasminogen at 2 µg/ml in PBS. Plasminogen binding was detected with primary rabbit anti-plasminogen anti-sera (Fitzgerald Industries) followed by secondary goat anti-rabbit IgG-HRP conjugated antibody (Sigma) and processed by autofluorography. For visualization and spot isolation of plasminogen-binding proteins, blots were stained with India Ink. Briefly, blots were washed two to three times in PBS, 0.3% Tween 20, and stained with India Ink in PBS, 0.3% Tween-20 with gentle agitation overnight. Blots were finally washed in several changes of PBS and rinsed in water to remove excess stain and detergent before excision and spot-analysis by mass spectrometry. Western Blot analysis: Electro-blotted PVDF membranes were first incubated overnight at 4°C in PBS with 1.5% BSA and 0.07% Tween 20 then exposed for 1 hr (25°C) to rabbit anti-human plasminogen (Fitzgerald Industries), diluted 1∶1000 in PBS+1.5% BSA. Membranes were washed four times with the same buffer and exposed to goat anti-rabbit HRP-conjugated IgG antibody (Sigma) diluted 1∶20,000 in PBS+1.5%BSA for 1 hr, washed 4× in PBS-T, followed by 5 min incubation in Immuno-Star HRP substrate (Bio-Rad) and detection by chemiluminescence.

### Immobilization of cell wall protein with plasminogen-conjugated sepharose beads

Lyophilized plasminogen was suspended in a solubilization buffer (10 mM PBS, pH 7.4, 0.5% octyl glucoside and 0.1% CHAPS) at 0.5 grams/ml and conjugated to CNBr-activated Sepharose 4B beads (Amersham) according to manufacturer's directions. Cell walls were prepared from 48 hr 50 ml YPD cultures as described above, suspended in solubilization buffer at 1.5 mg/ml protein then added to plasminogen-conjugated beads at a bead∶protein ratio 1∶2 (v/v) and tumbled for 1 hr at 25°C. Beads were isolated by 10,000 g sedimentation for 1 min, and washed three times in ice-cold solubilization buffer. Proteins were released by boiling in protein sample buffer, fractionated by SDS-PAGE and visualized by silver staining.

### Protein identification by LC-MS/MS

Silver-stained protein spots or bands were manually excised with a P2D1.5 pen (The Gel Company) from 2D and 1D gels and destained with a 1∶1 working solution of 30 mM potassium ferricyanide and 100 mM sodium thiosulfate. Excised gel spots were washed in 250 µl 100 mM ammonium bicarbonate (Ambic) for 20 min., 500 µl 50% acetonitrile (ACN): 50 mM Ambic for 1 hr, 50 µl ACN 10 min, then dried in a speed vacuum prior to trypsin digestion (10 µl 100 mM Ambic with 0.2 µg trypsin (Promega)) overnight at 37°C. Peptides were extracted twice in 150 µl 5% formic acid: 60% ACN for 1 hr. and pooled extracts were dried in a speed vacuum. Samples were suspended in 20 µl 0.5% formic acid: 3% acetonitrile in water and subjected to LC-MS/MS analysis. When necessary, protein spots were excised manually from India ink-stained (1 µl/ml India ink, 0.3% Tween-20 in PBS) blots pre-wet in methanol, destained in 50% methanol for 4 hrs, dried briefly, then digested with 14 µl 30% ACN: 50 mM Ambic containing 0.1 mg/ml trypsin, overnight at 37°C. Peptides were extracted with 50 µl 80% ACN by sonication for 15 min., dried in a speed vacuum, and resuspended in 20 µl 5% formic acid: 3% ACN prior to LC-MS/MS analysis.

The peptide digests were analyzed by multidimensional microscale capillary liquid chromatography coupled to an LTQ Proteome X mass spectrometer (Thermo Finnigan). Peptide mixtures (6.4 µl) were loaded from the autosampler onto a 300 µm×5 µm C18 trap column (LC Packings) at a rate of 30 µl/min. and eluted from the trap column with a 5–50% linear gradient of 0.5% formic acid in 80% ACN at a flow rate of 200 µl/min. The LTQ was run in positive ion mode with a 1.8 kV tip voltage and a capillary voltage of 4 V. Data were collected in a data-dependent mode with alternating MS scan survey over the mass range of 600–3500 and five MS/MS scans in exclusion dynamic mode. The spectra were obtained with a 2.5 *m/z* unit isolation window, relative collision energy of 35%, and a dynamic exclusion duration of 5 min. The SEQUEST algorithm within Bioworks rev3.3 (Thermo Finnigan) was used to search the resulting spectra against a database containing a subset of the Swiss-Prot and TrEMBL databases restricted for *Cryptococcus neoformans* (10,464 entries: 291 from Swiss-Prot, 10,173 from TrEMBL) with the following search parameters: peptide probabilities of less than 1.00e-3, two or more unique peptides, peptide Xcorr minimum values of 1.5 (+1 charge), 2.0 (+2 charge), and 2.5 (+3 charge). One missed trypsin cleavage was allowed. Permitted differential modifications included oxidation of methionines (+16 Da).

### Statistical analyses

Data are representative of at least three replicates and are expressed as means+/−SEM. A value of p<0.05 or less was considered statistically significant.

## Results

### Immobilization of plasminogen on the surface of *C. neoformans*

**Figure 1:**
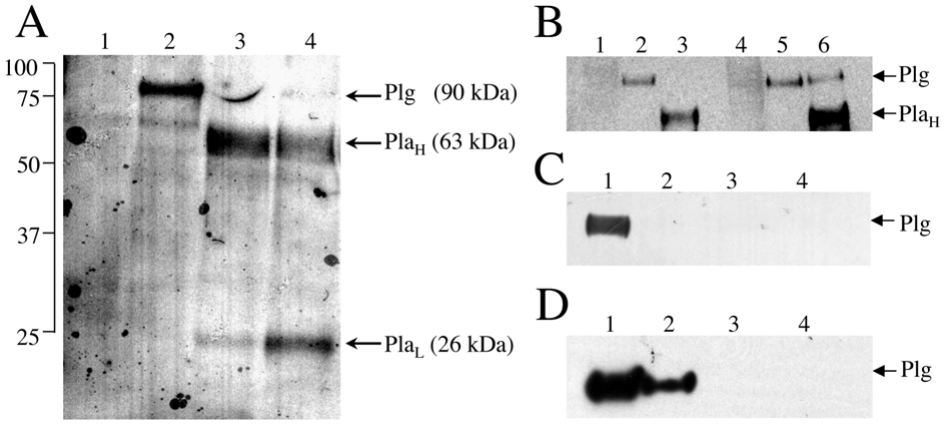
Plasminogen binds selectively and specifically to the cell-surface of intact *C. neoformans* strains. (A–B) Conversion of plasminogen (Plg) into plasmin heavy chain (Pla_H_) and light chain (Pla_L_) on the surface of intact *C. neoformans* serotype D and A strains. (A) Serotype D strain JEC21 was incubated in the presence or absence of plasminogen, tissue plasminogen activator (tPA), and/or the plasmin inhibitor aprotinin in phosphate-buffered saline with BSA. Cell wall proteins were released by boiling labeled cells in SDS-extraction buffer and fractionated by SDS-PAGE, transferred to PVDF, and Western blotted with polyclonal anti-plasminogen antibody. Lane descriptions as follow: cells (JEC21) only (1), 100 µg plasminogen (2), plasminogen and 100 ng tPA (3), plasminogen, tPA, and 1 unit aprotinin (4). (B) Serotype A strains C23 and A1 38-2 were incubated in the presence or absence of plasminogen and/or tPA for 4 hrs at 37°C prior to Western blot analysis as described above. Lanes: cells (C23) only (1), C23 with 15 µg plasminogen (2), C23 with plasminogen and 100 ng tPA (3), cells (A1 38-2) only (4), A1 38-2 with 15 µg plasminogen (5), and A1 38-2 with plasminogen and tPA (6). (C) Plasminogen associates with the cell wall of intact cells. Cells (1×10^10^) from log phase cultures (JEC21) were incubated 4 hr at 37°C in the presence (lane 1) or absence (lane 3) of 50 µg plasminogen and separated into cell wall and cytosol components, as described in Methods. Membranes (lane 2, 4) from cell walls were extracted and each fraction examined for the presence of plasminogen by Western blot analysis. Sample loading was uniform at 5 µg per well. (D) Sulfo-NHS-biotin and plasminogen compete for cell-surface binding sites. Log phase cells (JEC21) were initially labeled with sulfo-NHS-biotin in 0-, 1-, 10-, 100-fold molar equivalents of plasminogen then labeled 1 hr at 37°C with 50 µg plasminogen (lanes 1–4, respectively).

In order to determine the plasminogen binding potential of *C. neoformans*, we examined the ability of the JEC21 strain to both bind plasminogen on its surface and facilitate the tPA-induced activation of surface receptor-bound plasminogen to plasmin. Surface binding was assessed by incubating plasminogen (Glu-plasminogen) with intact log phase cells in the presence or absence of the plasminogen activator tPA and the plasmin inhibitor aprotinin prior to analysis of surface labeling by SDS-PAGE and Western blotting. Western blot analysis of labeled cell extracts with a polyclonal anti-plasminogen antibody showed that the single-chain plasminogen was bound to cells and was converted to active plasmin, as evidenced by the formation of the two-chain plasmin form, in the presence of tPA (Fig. 1A, lanes 3–4). There was no evidence of plasminogen conversion to plasmin in the absence of added tPA, indicating that the serotype D strains of *C. neoformans* do not express an endogenous plasminogen activator (Fig. 1A, lane 2). To ensure that the observed plasminogen labeling was a bona fide feature of *C. neoformans* and not specific to the JEC21 strain, duplicate reactions were performed for the related strains JEC20 and B3501A with identical results obtained for each (data not shown). Additionally, the inclusion of aprotinin, in the presence of Glu-plasminogen and tPA, prevented efficient plasmin-induced cleavage of the amino-terminal activation peptide (8 kDa) from the plasmin heavy chain (Pla_H_) and thus preventing the formation of Lys-plasminogen, which is the form of plasminogen most readily converted to active plasmin (Fig. 1A, lane 4) [63]. Analyses of supernatants from the labeling reactions failed to detect plasminogen conversion in the absence of cells (data not shown), demonstrating that surface receptor-mediated interactions are required for plasmin activation.

Because the serotype A strains of *C. neoformans* have greater clinical significance, we next examined plasminogen binding and activation in the serotype A isolates C23 and A1 38-2. Both are of the same genotype as strain H99 (A1/M1) and were obtained from clinical (C23) and environmental (A1 38-2) sources. These strains have been extensively characterized for their virulence phenotypes (provided by Dr. Anastasia Litvintseva), and both display comparable capsule sizes and levels of melanin production [56]. As we observed with the serotype D strains, the serotype A isolates examined also displayed plasminogen binding at the surface (Fig. 1B). Additionally, our results indicated that plasminogen conversion to plasmin for the serotype A isolates did not occur in the absence of added tPA, demonstrating that neither serotype (A or D) expresses an endogenous plasminogen activator (Fig. 1B, lanes 2, 5).

We next examined which surface-accessible structures (cell wall and/or plasma membrane) mediate plasminogen binding. Log phase cells were incubated with plasminogen and cell walls were isolated in the absence of detergent and purified, following extensive washing of the intact cells to remove unbound plasminogen, and tested for plasminogen labeling. As the plasma membrane and cell wall are tightly joined together, separation of the two organelles is likely to require detergent solubilization. Thus, to examine a possible role for plasma membrane-associated proteins in the binding of plasminogen to *C. neoformans*, isolated cell wall fractions were further treated with 0.5% Triton-X-100, and the membrane pellets recovered from the detergent-soluble fractions tested for plasminogen labeling. However, as indicated in Figure 1C, plasminogen labeling was found exclusively in the detergent-insoluble component of cell wall (Fig. 1C, lane 1), suggesting a minor role, if any, for membrane-bound proteins (Fig. 1C, lane 2) of cell wall-associated organelles, such as the plasma membrane, in plasminogen binding. The specificity of plasminogen detection was demonstrated by the absence of plasmingen labeling in cells that were not incubated with plasminogen prior to cell wall isolation (Fig. 1C, lanes 3–4).

Plasminogen has been observed to interact with a number of microorganisms by targeting the primary amine groups of cell-surface proteins. We therefore tested the ability of molecules or compounds that specifically target primary amines to inhibit plasminogen surface interactions. Biotinylation reagents are useful for this purpose because they target both α and ε- amine groups, thereby saturating all available cell-surface primary amines. The activity of the sulfonate derivative of the amine-reactive N-hydroxysuccinimide (NHS) ester of biotin used for these studies is membrane impermeable and thus confined to cell surfaces [59]. As shown in Figure 1D, cells that were first labeled with sulfo-NHS-biotin concentrations of 10-fold or greater than that of plasminogen failed to demonstrate plasminogen binding by Western blot analysis. In contrast, when the labeling-order was reversed so that cells received plasminogen prior to sulfo-NHS-biotin, cells retained surface-bound plasminogen for sulfo-NHS-biotin concentrations of up to 100-fold over that of plasminogen (Fig. S1). These latter findings may be anticipated given that plasminogen itself is rich in primary amines and thus capable of interacting with and quenching the effective concentration of sulfo-NHS-biotin applied in these experiments so that competitive binding with plasminogen does not occur, confirming the importance of *C. neoformans* cell-surface proteins in plasminogen recruitment.

### Plasminogen binding capacity of *C. neoformans* surface receptors

**Figure 2:**
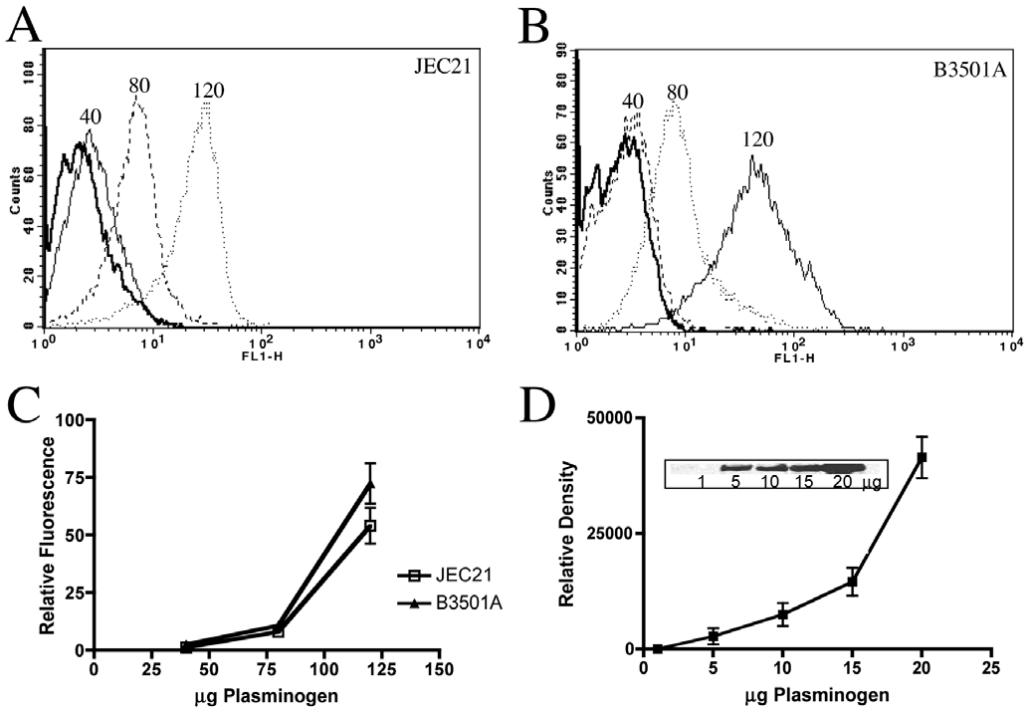
Plasminogen binding capacity of *C. neoformans* surface receptors. (A–B) Representative histograms for JEC21 (A) and B3501A (B) cultured at 25°C for 48 hr in 50 ml YPD. Cells were suspended at 10^7^/ml and labeled for 1 hr at 37°C with 40 µg (solid line), 80 µg (dashed line), or 120 µg (dotted line) plasminogen, followed by exposure to rabbit anti-human plasminogen antiserum and FITC-conjugated secondary antibody. The numbers located above each curve in the histograms indicate plasminogen-labeling concentration (µg) for corresponding populations. Each histogram shows cell number as a function of relative fluorescence obtained for a total of 10,000 events per population. The control population (bold solid line) was treated with primary and secondary antibody in the absence of plasminogen labeling. Greater than 90% of the cells examined stained positive for plasminogen under the growth conditions applied, so gating was not necessary. Each data set is representative of three independent experiments. (C) Plasminogen binding curves for JEC21 (squares) and B3501A (closed triangles) cultured and plasminogen-labeled as in (A–B), averaged from six independent experiments. Data were adjusted for nonspecific binding, which is represented by the baseline. (Kd JEC21 = 900 nM, Kd B3501A = 750 nM). (D) Plasminogen binding curve of JEC21 as detected by Western blot analysis from three independent experiments. Cells were incubated with the indicated concentrations of plasminogen for 1 hr 37°C then examined for surface-bound plasminogen by Western blot analysis. The graph shows the relative signal density detected for the plasminogen concentrations indicated on the abscissa. A representative blot is shown with plasminogen concentrations (µg) concentrations indicated below each band.

The binding of *C. neoformans* to plasminogen was quantitatively examined by flow cytometry and SDS-PAGE analysis. Both approaches were used in this study to obtain an approximate measure of ligand binding sensitivity and the relative limits of plasminogen binding capacity. Flow cytometry histograms consistently showed that ≥95% of (log phase) cell populations stained positive for plasminogen. Surface plasminogen first became detectable at plasminogen concentrations of 40 µg (∼10 µM) for strains JEC21 (Fig. 2A) and B3501A (Fig. 2B) and was observed as a slight shift in signal intensity over control populations not labeled with plasminogen. A maximal shift in signal intensity occurred with 120 µg (∼3 µM) plasminogen under the experimental conditions applied. Of the two strains examined in these experiments, B3501A consistently showed a greater binding capacity for plasminogen by flow cytometry analysis, but this difference was not significant and was evident only at plasminogen-labeling concentrations of 80 µg or above (Fig. 2C).

We observed relatively high autofluorescence values for all *C. neoformans* strains tested by flow cytometry that may have in turn artificially elevated the range of plasminogen concentrations required for label detection and characterization. We therefore used SDS-PAGE and Western blotting to additionally examine the plasminogen-binding activity of strains JEC21 and B3501A. Under plasminogen labeling conditions similar to those applied during flow cytometry, we found that surface plasminogen could be detected at plasminogen labeling concentrations as low as 5 µg (100 nM; Fig. 2D), which was ∼10-fold lower than the minimal labeling concentrations required for demonstrable binding by flow cytometry analysis (Fig. 2C). Progressive increases in labeling concentration resulted in correspondingly higher signal intensities up to a maximal intensity with 80 µg plasminogen exposure (data not shown). This latter value was ∼30% lower than the maximal labeling concentration observed by flow cytometry analysis for similar numbers of cells. A 10-fold increase in the number of cells per labeling reaction to 1×10^8^ resulted in a corresponding decrease in the minimal concentration of bound plasminogen that could be detected on intact cells by SDS-PAGE/Western blot to 10 nM (data not shown).

**Figure 3:**
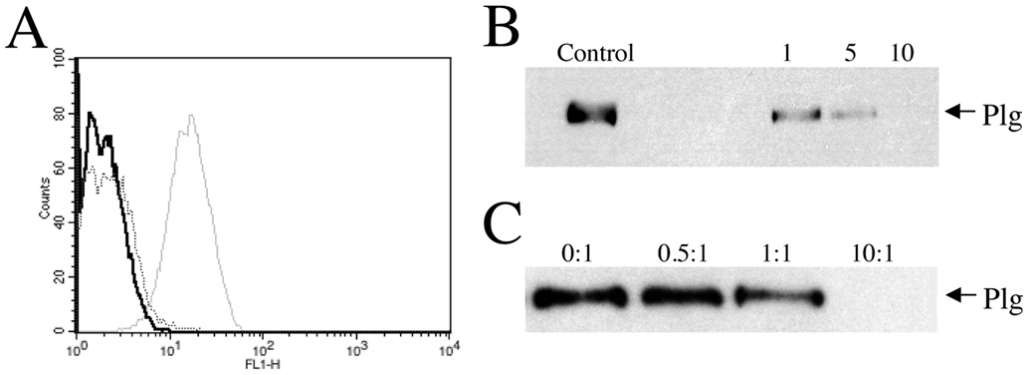
Surface-exposed lysines are required for plasminogen binding. (A–B) Influence of carboxypeptidase B pretreatment on plasminogen binding. Cells were grown to log phase at 25°C and incubated 30 min at 37°C in the presence of carboxypeptidase B prior to a subsequent incubation with 50 µg plasminogen for 1 hr at 37°C and examination by either: flow cytometry (A), or Western blot analysis (B). In (A), the control population, indicated by a solid black line, was treated with primary and secondary antibody in the absence of plasminogen labeling. The dashed line represents cells incubated in the absence of carboxypeptidase (0 units) prior to plasminogen labeling (50 µg), while the population depicted by the solid gray line was pretreated with 10 units of carboxypeptidase prior to the addition of 50 µg plasminogen. In (B), carboxypeptidase treatments (in units), prior to the addition of plasminogen, are labeled as control (0 U), 1 (1 U), 5 (5 U), and 10 (10 U) above each corresponding sample lane. Plasminogen incubation for 1 hr at 37°C was followed by extensive washing and subsequent Western blot analysis of plasminogen binding. (C) Effects of εACA pretreatment on plasminogen binding. Cells were incubated for 30 min at 37°C with εACA at 0, 0.5, 1, or 10 molar equivalents in excess of the concentration of plasminogen used (20 µg, ∼400 nM). Plasminogen was added after the initial εACA incubation, followed by an additional incubation for 1 hr at 37°C and subsequent Western blot analysis of plasminogen binding. The molar ratios of εACA∶plasminogen are noted above the lanes for each corresponding sample. The data shown are representative of three independent experiments.

Given the crucial role of free surface α- and/or ε-amine groups in mediating the plasminogen interactions with *C. neoformans*, we further examined the effect of agents that selectively inhibit ligand interactions with cell wall protein carboxy-terminal amine groups using either competitive antagonism with the lysine analog εACA, or the targeted cleavage of C-terminal lysines with carboxypeptidase B. The results shown in Figure 3 indicate a major role for surface amine groups in facilitating surface protein-plasminogen interactions. Cells pre-treated with carboxypeptidase B (Fig. 3A and B) or 10-fold excess εACA (Fig. 3C) were no longer able to bind plasminogen, as determined by both flow cytometry (Fig. 3A) and Western blot analysis (Fig. 3B and 3C). Accordingly, these data suggest a facilitating role for both internal and C-terminal lysines residues present on surface proteins in plasminogen recruitment by *C. neoformans*.

### Influence of capsule on plasminogen binding and activation

**Figure 4:**
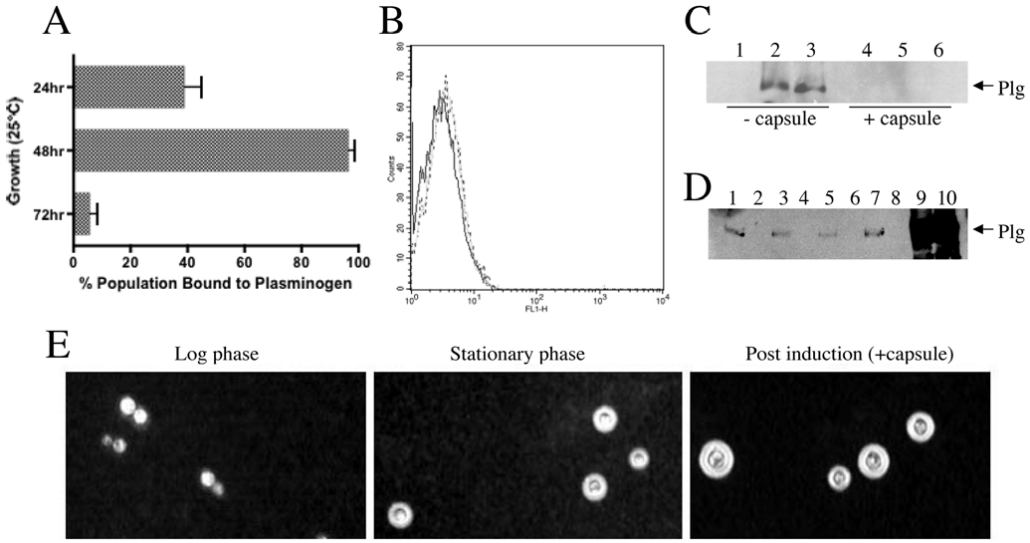
Effects of growth phase and capsule development on plasminogen binding activity of *C. neoformans*. (A) Plasminogen binding capacity at distinct stages of cell growth in YPD culture media. Serotype D strain JEC21 was incubated for the times indicated in 50 ml YPD and measured by flow cytometry for the ability to bind plasminogen. The data shown were quantified from flow cytometry histograms as the percent plasminogen binding over control (abscissa) for each time point described (ordinate) and are representative three independent experiments. The 24, 48 and 72 hr time-points indicated correspond to lag, log and stationary growth phases, respectively. (B–D) Plasminogen binding activity of encapsulated cells. Flow cytometry histogram (B) and PAGE/Western blot (C) showing little or no plasminogen binding activity for encapsulated JEC21 cells compared to reduced capsule (uninduced and DMSO-treated) controls. Strain JEC21 was grown in either YPD (C; lanes 1–3) or capsule induction (C; lanes 4–6) medium prior to labeling for: 1 hr (B) or 4 hr (C) at 37°C with 120 µg (B, broken line) or 100 µg (C; lanes 2–3, 5–6) plasminogen. Lanes 3 and 5 of (C) were treated 1 hr with DMSO prior to receiving 100 µg plasminogen. Control cells (B, bold line; C, lanes 1 and 4) received primary and secondary antibody in the absence of plasminogen labeling. (D) Western blot analysis of plasminogen binding activity for serotype D strains JEC21, FCH79 (*CAP59 cap59::nat*), FCH78 (*cap59::nat*), and the serotype A strains C23 and A1 38-2. Cells were grown in YPD (−cap) or capsule induction (+cap) medium, labeled with plasminogen, and subjected to Western blot analysis, as described above, Lanes: JEC21 −cap (1), JEC21 +cap (2), FCH79 −cap (3), FCH79 +cap (4), C23 −cap (5), C23 +cap (6), A1 38-2 −cap (7), A1 38-2 +cap (8), FCH78 −cap (9), and FCH78 +cap (10). (E) Examination of capsule formation. Aliquots of strain JEC21 were examined at log or stationary growth phases, or after incubation in capsule induction medium, and examined for capsule by India ink staining at 40× magnification. Results are averaged (A) from three independent experiments or representative of either two (D) or three experiments (B–C).

The surface-accessibility of wall-associated proteins, which serve as receptors for binding of plasminogen in *C. neoformans*, may change as cells age during culture, *in vitro*. Culture-dependent changes in nutrient availability could, for example, modify plasminogen binding activity through associated changes in cell wall protein expression or their surface accessibility. We therefore examined the plasminogen-binding ability of cells during lag (24 hr) and stationary (72 hr) phases, using log phase (48 hr) cells as a standard for comparison. Cells at different growth stages were compared in parallel for relative plasminogen binding following exposure to 120 µg (∼3 µM) plasminogen, the previously observed maximum labeling concentration bound by log phase cells in our experimental conditions (Fig. 2). Signal intensities from flow cytometry histograms were quantified and presented in the bar graph shown in Figure 4A, which indicates that a significant variance in plasminogen binding does occur as cells progress through different stages of growth. Notably, the highest plasminogen binding was observed for log phase (48 hr) cells (Fig. 4A). In contrast, cells harvested during lag phase growth demonstrated less than half the plasminogen binding activity of log phase cells, while stationary phase cells exhibited a greater than 90% relative decrease in plasminogen binding activity compared to log phase cells (Fig. 4A).

As plasminogen recruitment and the plasminogen-activated fibrinolytic system have been implicated in facilitating microbial pathogenesis, we next examined the influence of capsule formation on the cell wall surface accessibility and plasminogen binding capacity of the serotype D strains JEC21, FCH78 (*cap59::nat*), and FCH79 (*CAP59 cap59::nat*) and the serotype A isolates C23 and A1 38-2. Cell growth of each strain in a PBS-diluted Sabouraud capsule induction medium, with the exception of the *cap59Δ* isolate, resulted in robust capsule formation, as shown for strain JEC21 (Fig. 4E), validating the effectiveness of the capsule induction protocol. Post-induction encapsulated strains were next examined for plasminogen binding activity by SDS-PAGE/Western blot analysis. Encapsulated serotype A and D cells, incubated in the presence of plasminogen, showed no plasminogen binding by Western blot analysis (Fig. 4C, lane 5 and Fig. 4D, lanes 2, 4, 6, and 8) as compared to uninduced (hypocapsular) (Fig. 4C, lane 2 and Fig. 4D lanes 1, 3, 5, and 7) or genetically-derived acapsular (*cap59Δ*) cells (Fig. 4D, lanes 9–10), which were positive for plasminogen label. Interestingly, the relative plasminogen binding activity of the acapsular *cap59Δ* strain was considerably more pronounced than that of the hypocapsular (uninduced) controls. These results may indicate that the changes in surface density associated with capsule formation could mask or otherwise interfere with the accessibility of proteins to plasminogen.

As treatment with DMSO has been shown to remove capsular polysaccharides from encapsulated cells [64], [65], we also examined the potential for masked plasminogen binding on the cell wall surface in the presence of intact capsule. Following incubation of cells (encapsulated or the uninduced controls) with plasminogen, washed cells were exposed to DMSO prior to Western blot analysis of surface plasminogen labeling. Although DMSO-treatment resulted in extensive capsule removal, as observed by India Ink staining (data not shown), this treatment did not reveal detectable plasminogen-binding on the underlying cell wall in encapsulated cells (Fig. 4C, lane 6). Additionally, DMSO treatment did not disrupt detection of plasminogen labeling on the surface of uninduced (hypocapsular) cells (Fig. 4C, lane 3).

When encapsulated cells from strain JEC21 were examined for plasminogen binding activity by flow cytometry, only a minor shift could be detected in response to 120 µg plasminogen (Fig. 4B) relative to log phase cells receiving the same amount of label (Figs. 2A–B). Indeed, we found the minor shifts in the plasminogen binding activity of both encapsulated and stationary phase cells to be quantitatively similar. When we examined stationary phase cells microscopically, prominent capsules were observed in strain JEC21 (Fig. 4E). These data, together with results from Figures 4C and D, suggest that capsule formation may occlude or otherwise constrain the presentation of cell wall proteins and inhibit the interaction of plasminogen with surface receptors, although factors other than capsule formation may compromise the ability of stationary phase cells to interact with plasminogen.

### Proteomic analysis of plasminogen-binding cell wall proteins

**Figure 5:**
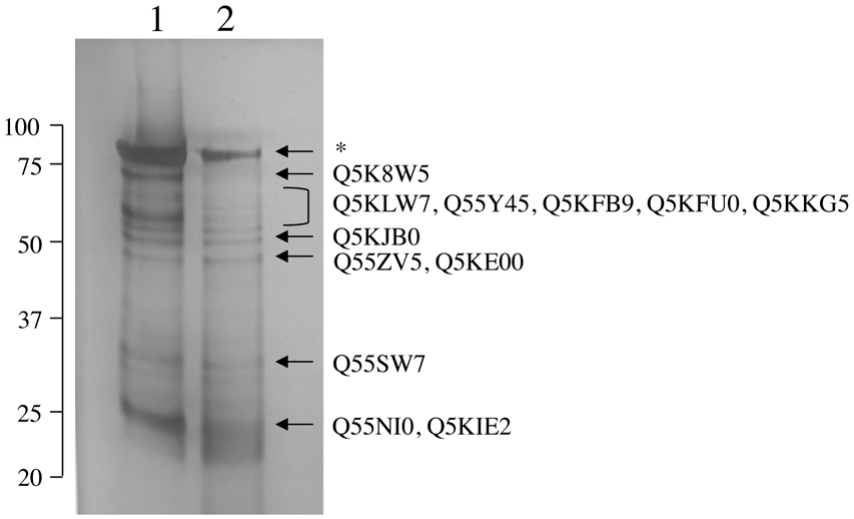
Identification of plasminogen-binding cell wall proteins by 1D-PAGE and LC-MS/MS. Precipitation of purified cell wall protein preparations made from strain B3501A with plasminogen-conjugated CNBr-sepharose beads. Protein profiles obtained from bead eluate fractions (lane 1 = fraction 1, lane 2 = fraction 2) are compared after silver staining of 10% SDS-PAGE gels. Molecular weights are indicated on the left. The data shown is representative of three experiments. Indicated are the positions of identified plasminogen-binding proteins. Identified proteins are listed in Table S1.

The observation of surface-associated binding and activation of plasminogen to plasmin on the *C. neoformans* cell wall led us to investigate the identity of the cell wall proteins that function as receptors for plasminogen. An affinity chromatography approach was used to isolate plasminogen-binding cell wall proteins from purified cell wall-associated protein fractions. Cell wall protein extracts were incubated with plasminogen-bound or unbound CNBr-sepharose beads and profiles of proteins recovered from both the bead material and wash supernatants were visualized by silver staining of SDS-PAGE gels after 1D separation. Silver staining revealed similar patterns for both B3501A and JEC21 strains in the presence of plasminogen (Fig. 5, data not shown). In the absence of plasminogen, the bead pull down of cell wall protein extracts failed to yield detectable protein. Bands were excised from the silver stained gel and digested with trypsin to release peptides for LC-MS/MS. Data from mass spectrometry was filtered and searched with the SEQUEST algorithm against a database containing a subset of the Swiss-Prot and TrEMBL databases restricted for *Cryptococcus neoformans*, LC-MS/MS analysis of the proteins recovered from plasminogen-CNBr beads suggested that a number of cytoplasmic proteins are located on the surface of *C. neoformans*, where they play a role in plasminogen recruitment, in vivo. The proteins identified from CNBr-plasminogen eluates included functionally diverse proteins, such as heat shock proteins 60 and 70, pyruvate kinase, phosphoglycerate kinase, the ATP synthase alpha and beta subunits, and the response to stress-related protein (Q5KJB0) (Table S1). Three of the twelve proteins, including glucose-6-phosphate isomerase, phosphoglycerate kinase, and the putative uncharacterized protein Q55ZV5, are predicted to have carboxyl-terminal lysine residues.

**Figure 6:**
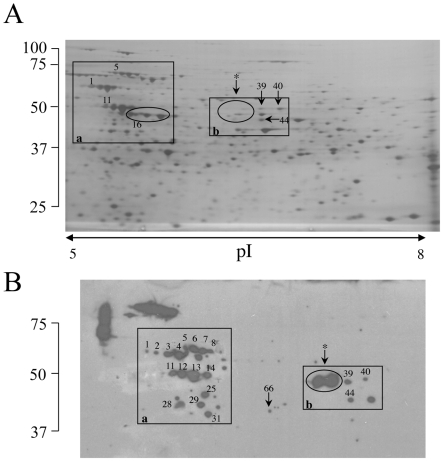
Identification of plasminogen-binding cell wall proteins of *C. neoformans* by 2D-PAGE and LC-MS/MS. Two-dimensional gel electrophoretic characterization of the cell wall proteome from a silver-stained gel (A), and the corresponding plasminogen-binding proteins (B) by ligand (plasminogen) overlay and western blot analysis with anti-plasminogen antibody. Indicated are the positions of identified plasminogen-binding proteins. Identified proteins shown in (B) are listed in Table S2. (* indicates spots sequenced following excision from the PVDF membrane due to lack of detection by silver-staining).

**Figure 7:**
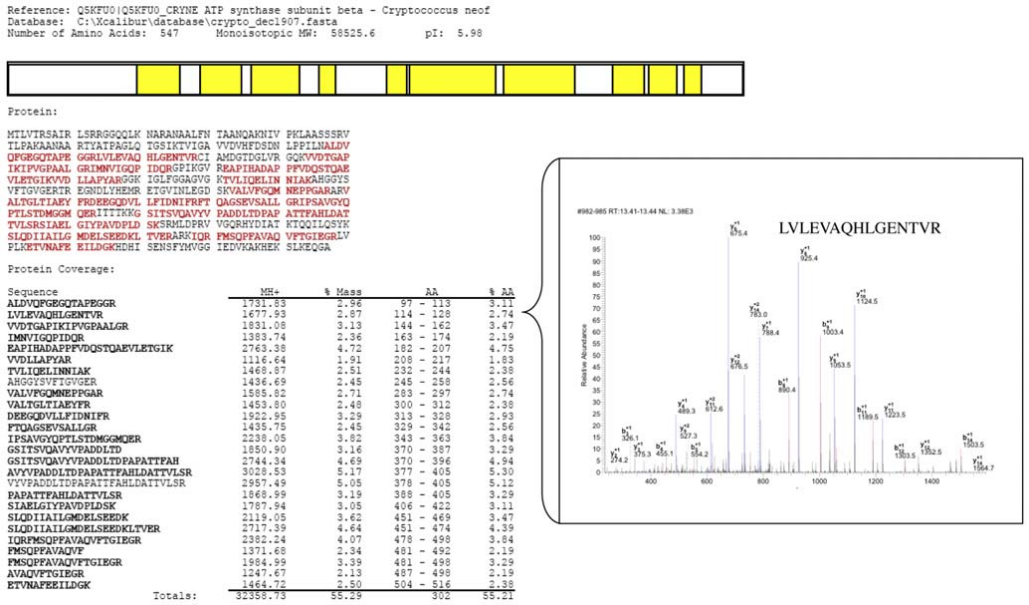
Identification of spot 12 as Q5KFU0, an ATP synthase beta subunit. Overview of identification is shown. Shaded areas (yellow in graphical display and red for protein sequence) indicate peptide coverage. Shown as an inset is a representative MS/MS spectra for peptide [LVLEVAQHLGENTVR] from Q5KFU0.

We next sought to both confirm and to identify additional plasminogen-associated surface receptors using a combination of ligand affinity and proteomics. Cell surface-associated proteins, which include noncovalently-bound and disulfide bridge-associated proteins, were isolated from cell wall fractions by SDS-extraction and analyzed by two-dimensional gel electrophoresis and silver staining [60], [61]. The method of cell wall protein isolation used in our study was chosen to enrich for the identification of the subpopulation of cell wall proteins that facilitate plasminogen binding through receptor-mediated interactions, which include predominantly “atypical” or “non-classical” cell wall proteins of cytosolic origin. Duplicate gels were transferred to PVDF for ligand binding with plasminogen to identify cell wall-associated plasminogen binding receptors and the results compared to the protein pattern of the silver stained counterpart (Fig. 6). Spots that corresponded to plasminogen-binding proteins, as well as selected spots to serve as references, were excised from the silver stained gel and digested with trypsin to release peptides for LC-MS/MS. Additional spots were excised from the PVDF membrane and similarly processed for both additional data confirmation and if corresponding spots were not detectable on the matching silver stained gel. As before, spectral data was searched against a database restricted for *C. neoformans*, facilitating the identification of ten plasminogen-binding proteins and three additional reference proteins, listed in Table S2. While four of the identified proteins; Hsp60 and 70, the ATP synthase beta subunit, and phosphoglycerate kinase; were also found with the affinity chromatography approach (Fig. 5, Table S1), there were six additional proteins identified, including transaldolase, fructose-bisphosphate aldolase, and glutamate dehydrogenase (Table S2). Figure 7 and Table S3 show examples of MS/MS data output and peptide quality parameters for the protein identified from spot 12 (Q5KFU0, ATP synthase beta subunit). Of the ten plasminogen-binding proteins identified with the ligand affinity method, only one (phosphoglycerate kinase) was predicted to contain a carboxyl-terminal lysine, suggesting that plasminogen binding to a majority of the surface receptors could be mediated in part by internal lysine residues, as demonstrated by the inhibition of plasminogen binding by the lysine analog εACA (Fig. 3C). Comparisons of the spot intensities between the silver-stained gel and the corresponding ligand blot show that glutamate dehydrogenase (Q5KL32, indicated by * in Fig. 6B) exhibited the highest relative affinity for plasminogen among the reactive spots, even though it was not detectable in the silver-stained 2D gel. Also observed among the plasminogen-binding proteins was the linear pattern of spots with the same MW but different isoelectric point (pI) values, a pattern characteristic of isoforms that result from differential post-translational protein modifications (Fig. 6). Proteins with this feature included the heat shock proteins (Hsp60 and 70), as well as the ATP synthase beta subunit. Additionally, the reference protein enolase, which did not bind plasminogen in this study despite the presence of a carboxyl-terminal lysine residue, also displayed the isoform cluster typically observed for this protein.

### Plasminogen-mediated ECM degradation

**Figure 8:**
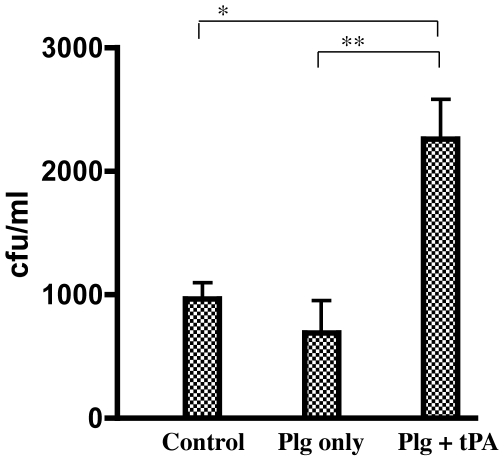
Penetration of *C. neoformans* through reconstituted ECM. The ECM invasion chambers are composed of matrigel (basement membrane) layered on membranes with 8 µm pores. Strain JEC21 was incubated with plasminogen in phosphate-buffered saline with BSA in the presence or absence of tissue-derived plasminogen activator (tPA), incubated in the upper chamber of the transwell for 24 hours at 37°C prior to analysis of colony counts from the lower well (* (p = 0.0093); ** (p = 0.0084)).

We next examined the ability of surface-bound plasmin to facilitate penetration of *C. neoformans* through Matrigel, a reconstituted extracellular matrix (ECM) preparation used in BioCoat Matrigel invasion chambers, an in vitro system for the study of cell invasion through basement membrane, consisting of cell culture inserts containing an 8 µm pore-size PET membrane coated with a uniform layer of Matrigel. Log phase JEC21 cells (1×10^8^) were incubated in the presence or absence of plasminogen and/or tPA, then washed to remove unbound plasminogen and added to the upper chamber of the transwell. The invasive potential of JEC21 was determined by calculating colony-forming units from medium in the lower chamber following 22 hrs of incubation. Controls included cells that were not labeled with plasminogen, as well as the omission of the plasminogen activator tPA. In the presence of surface-bound active plasmin, *C. neoformans* displayed detectable ability to penetrate the Matrigel into the lower chamber compared to cells without bound plasminogen or active plasmin (Fig. 8). This difference was statistically significant (P<0.01) for the plasmin-labeled replicates compared to unlabeled or plasminogen-only controls and suggests that *C. neoformans* may utilize plasmin, a potent serine protease, to facilitate its invasive potential across extracellular matrices.

## Discussion

The host plasminogen system is frequently exploited by pathogenic organisms, both bacterial and fungal, to promote tissue invasion and disease. In this study, we have shown that *C. neoformans* binds plasminogen at its surface, facilitating the activation of plasminogen to the broad-specificity serine protease plasmin. Most significantly, we have also demonstrated the importance of surface-bound activated plasmin in the invasion of reconstituted extracellular matrix (ECM), in vitro, by *C. neoformans*.

We found that plasminogen binding to *C. neoformans* was strictly cell wall-associated, with specific labeling demonstrated for the surface of intact cells, as well as isolated cell wall fractions. The binding of plasminogen was concentration-dependent for log phase cells with affinities for both JEC21 and B3501A comparable to those reported for *C. albicans* and bacterial pathogens such as the *Neisseria* and *Streptococcal* species [44]. An intriguing observation was the observed difference in the affinities of JEC21 and B3501A for plasminogen. The observed dissociation constant (Kd) for JEC21 was 900 nM, while the Kd for B3501A was found to be 750 nM, suggesting that the B3501A strain had a higher affinity for plasminogen. While the significance of this finding is not known, it may partially explain the differential virulence properties associated with these two genetically similar strains [66].

The binding of plasminogen to the surface of *C. neoformans* was found to be lysine-dependent, a feature that is common to all plasminogen-binding species [41], [49], [67]. Treatment of *C. neoformans* with basic carboxypeptidase, which cleaves exposed C-terminal lysine residues, prevented surface-associated plasminogen binding. Similar results were obtained following treatment with the lysine analog εACA. Together, these results suggest that both internal and C-terminal lysine residues present on the surface of C. *neoformans* contribute to plasminogen recruitment and indicate that the plasminogen receptor repertoire of *C. neoformans* is composed of a diverse protein population.

The ability of *C. neoformans* to facilitate the conversion of bound plasminogen to the serine protease plasmin was found to be conserved among the serotype A and D strains tested, suggesting that the observed virulence differences among the two serotypes, as well as among strains within each serotype (JEC20, JEC21, and B3501A for serotype D; and C23 and A1 38-2 for serotype A), are independent of the ability to bind and activate plasminogen [66]. Additionally, the conversion of plasminogen to plasmin on the surface of *C. neoformans* was dependent on the presence of the exogenous plasminogen activator tPA, demonstrating that *C. neoformans* does not express a functional plasminogen activator.

The cryptococcal capsule is a significant virulence determinant and serves to protect microorganisms from phagocytosis during infection [68]–[71]. Since capsule formation may occlude or otherwise constrain the presentation of cell wall proteins and inhibit the interaction of plasminogen with surface receptors, we compared the plasminogen-binding activities for capsular, hypocapsular, and acapsular cells. As might be expected, the absence of capsule promoted robust plasminogen labeling, while the presence of capsule was sufficient to prevent plasminogen binding at the cell wall surface. However, factors that influence cell wall composition during capsule synthesis, rather than the presence of capsule alone, may further compromise the ability of encapsulated cells to interact with plasminogen.

While capsule formation is necessary for the development and persistence of cryptococcal infection, there are several events during the infection cycle in which the acapsular or minimally encapsulated state would be advantageous, particularly during hematogenous dissemination and endothelial cell interaction [1], [72]–[74]. Therefore, we propose that *C. neoformans*, like other encapsulated organisms, including *Neisseria meningitides* and *Streptococcus pneumoniae*, which utilize plasminogen recruitment to promote infection, may also exploit the host plasminogen system during selected phases of the infection cycle, such as dissemination and tissue invasion, when encapsulation is not as favorable [75], [76]. Finally, our results suggest that growth-related changes in cell wall protein expression, as well as stress/virulence-related changes in the topology of surface protein presentation, may dynamically modulate the ability of this organism to interact with and recruit plasminogen and possibly other plasma-derived mammalian factors implicated in the progression of microbial disease, *in vivo*.

Analyses of the plasminogen-binding protein population by both affinity chromatography and ligand binding permitted the identification of several surface proteins that serve as plasminogen receptors in *C. neoformans*. These include two proteins, phosphoglycerate kinase (Pgk) and fructose bisphosphate aldolase (Fba), previously identified as plasminogen-binding receptors in *C. albicans*, as well as several proteins, including Hsp70, the ATP synthase alpha and beta subunits, and glutamate dehydrogenase, all of which have been previously reported to be localized to the cell wall and/or cell wall transport vesicles (virulence bags) in *C. neoformans* [44], [77]–[79]. While the presence of cytosol-derived proteins within the fungal cell wall has been extensively described, the method by which these “moonlighting” proteins become incorporated into the cell wall has not been established, as they generally lack the classical signal sequences necessary for secretion (reviewed in [25], [80]) [61], [81]. Although alternative secretion pathways, adventitious binding, or cytosolic contamination have all been suggested as possible explanations for the presence of cytosolic proteins within the cell wall of various fungi, our findings clearly show the specific localization of the plasminogen-binding receptors within the cell wall of *C. neoformans*. The additional discovery that several of these receptors, while of cytosolic origin, are also found in cell wall transport vesicles or “virulence bags” suggests that *C. neoformans* manifests a complex secretion mechanism that may facilitate the delivery of atypical cell wall proteins, as well as other pathogenesis-related molecules [77].

Our findings also demonstrate the cell wall association of the multifunctional protein enolase, a predominant plasminogen-binding and cell wall incorporated protein in *C. albicans*, *A. fumigatus*, and *P. jiroveci* (*P. carinii*) [45], [46], [82]–[87]. Although our results did not corroborate a role for the importance of enolase in plasminogen-binding in *C. neoformans*, we surmise that, due to the presence of a C-terminal lysine and its relative abundance in the cell wall of *C. neoformans*, enolase is indeed likely to contribute to plasminogen binding, and our inability to detect binding was caused by the type of plasminogen used in the ligand binding studies, as the affinity of Lys-plasminogen for C-terminal lysines is substantially higher than that of the Glu-plasminogen we utilized [63].

Functional studies to address the significance of surface-associated plasminogen binding in the invasiveness of *C. neoformans* demonstrated that plasmin-coated organisms possess an increased potential to penetrate extracellular matrix, in vitro. Similar results demonstrating the importance of plasminogen-binding have been observed for other fungal pathogens. Most notable are the recent studies demonstrating that susceptibility to invasive aspergillosis is strongly influenced by the host plasminogen system and that plasminogen activation on the surface of both *A. fumigatus* and *C. albicans* promotes extracellular matrix invasion [46], [48]. Although multiple factors contribute to fungal virulence, including the expression of extracellular proteases, morphogenic switching, adherence, hydrolytic enzymes, and capsule production, the conserved ability of fungal pathogens to subvert the host plasminogen system suggests that plasminogen binding may be an additional mechanism used by fungi to promote dissemination and tissue invasion during infection [27], [46], [48], [88], [89].

In summary, we have shown that *C. neoformans* may utilize the host plasminogen system to cross tissue barriers, providing support for the hypothesis that plasminogen-binding could contribute to the invasion of the blood-brain barrier by penetration of the brain endothelial cells and underlying matrix. In addition, we have identified the cell wall-associated proteins that serve as plasminogen receptors and characterized both the plasminogen-binding and plasmin-activation potential for this significant human pathogen. The results of this study provide evidence for the cooperative role of multiple virulence determinants in *C. neoformans* pathogenesis and suggest new avenues for the development of anti-infective agents in the prevention of fungal tissue invasion.

## Supporting Information

Figure S1 Plasminogen binds selectively and specifically to the cell-surface of intact *C. neoformans* strain JEC21. Cells were labeled in reverse-order to Figure 1C, with cultures first labeled with 50 µg plasminogen, prior transferred to ice and incubation with sulfo-NHS-biotin at 1-, 10-, 100-, 500-, or 1000-fold equivalent of added plasminogen (lanes 1–5, respectively). Western blots of cell wall proteins were prepared and first examined for plasminogen binding (a) then stripped and re-examined for biotin (b). Arrows indicate location of 90 kDa marker. Lane numbers are noted above each gel. Each data set is representative of three independent experiments. Similar results were obtained for strains JEC20 and B3501A.
(0.15 MB TIF)

Table S1 Distinct bands (shown in Fig. 5) were excised, subjected to trypsin digestion, LC-MS/MS analysis, and MS/MS spectra analysis to identify each excised protein. Peptides were searched against the *C. neoformans* protein database and results were filtered according to peptide probabilities, SEQUEST X-corr (XC) scores, peptide charge state, and two or more unique peptide hits per protein to eliminate false or low-quality identifications. Locus ID, protein name, protein molecular weight (MW), Sequest score (XC), percent sequence coverage, and number of peptides identified amino are indicated for each. * The affinity ligand plasminogen is included as an additional control for peptide identification following spot excision.
(0.03 MB XLS)

Table S2 Plasminogen-binding spots and nonbinding controls (spots shown in Figure 6) were excised, subjected to trypsin digestion, LC-MS/MS analysis, and MS/MS spectra analysis to identify each excised protein. Peptides were searched against the *C. neoformans* protein database and results were filtered according to peptide probabilities, SEQUEST X-corr (XC) scores, peptide charge state, and two or more unique peptide hits per protein to eliminate false or low-quality identifications. Locus ID, protein name, protein molecular weight (MW), isoeletric point (pI), Sequest score (XC), percent sequence coverage, and number of peptides identified amino are indicated for each. Scores, coverage and # peptides are listed for a single spot within an isoform cluster of identical proteins. Plasminogen-binding proteins listed in upper portion of table with plasminogen-binding negative listed in lower portion (below line).
(0.03 MB XLS)

Table S3 Reference scan (Rscan), peptide sequence, mass to charge ratio in positive ion mode (MH+), peptide charge (z), peptide probability (PP), and Sequest score (X-corr).
(0.67 MB TIF)
