# Supplementary figures and images for: Surface-Associated Plasminogen Binding of Cryptococcus neoformans Promotes Extracellular Matrix Invasion
Source: PLoS One. 2009 Jun 3;4(6):e5780. doi: 10.1371/journal.pone.0005780 (PMC2685986; doi:10.1371/journal.pone.0005780)

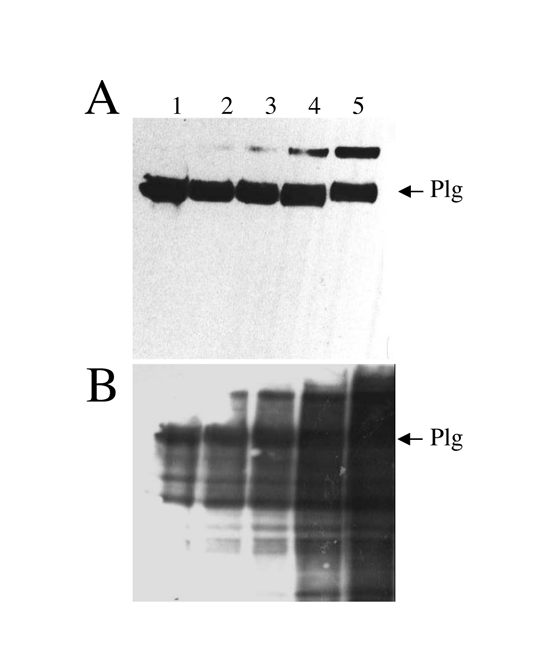

Supplement: Figure S1 — Plasminogen binds selectively and specifically to the cell-surface of intact C. neoformans strain JEC21. Cells were labeled in reverse-order to Figure 1C, with cultures first labeled with 50 µg plasminogen, prior transferred to ice and incubation with sulfo-NHS-biotin at 1-, 10-, 100-, 500-, or 1000-fold equivalent of added plasminogen (lanes 1–5, respectively). Western blots of cell wall proteins were prepared and first examined for plasminogen binding (a) then stripped and re-examined for biotin (b). Arrows indicate location of 90 kDa marker. Lane numbers are noted above each gel. Each data set is representative of three independent experiments. Similar results were obtained for strains JEC20 and B3501A. (0.15 MB TIF) [file pone.0005780.s001.tif]

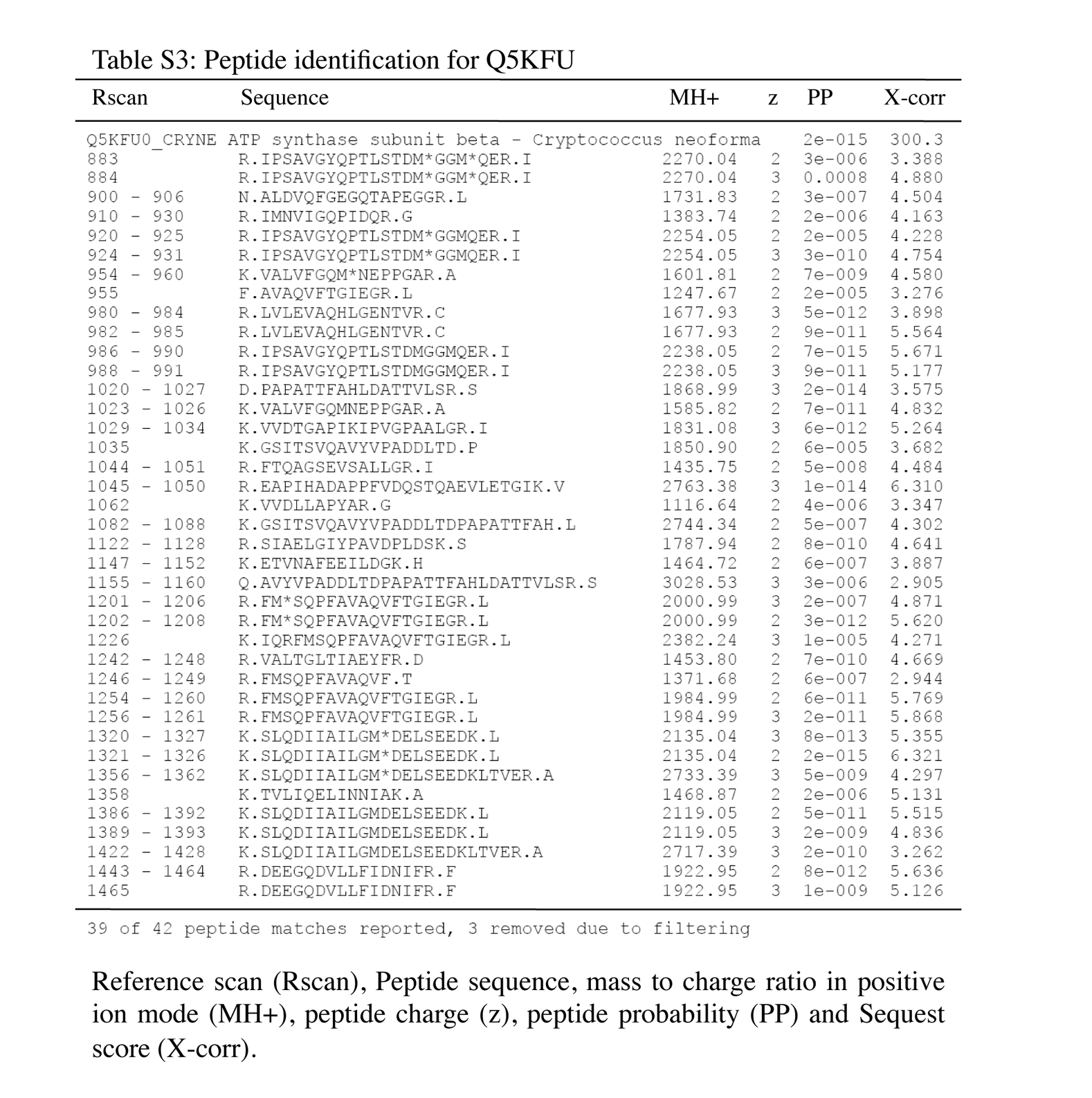

Supplement: Table S3 — Reference scan (Rscan), peptide sequence, mass to charge ratio in positive ion mode (MH+), peptide charge (z), peptide probability (PP), and Sequest score (X-corr). (0.67 MB TIF) [file pone.0005780.s004.tif]
